# Independent and joint effects of genomic and exposomic loads for schizophrenia on psychotic experiences in adolescents of European ancestry

**DOI:** 10.1038/s41537-025-00569-2

**Published:** 2025-02-22

**Authors:** Matteo Di Vincenzo, Thanavadee Prachason, Gaia Sampogna, Angelo Arias-Magnasco, Bochao Danae Lin, Lotta-Katrin Pries, Jim van Os, Bart P. F. Rutten, Ran Barzilay, Andrea Fiorillo, Sinan Guloksuz

**Affiliations:** 1 Department of Psychiatry, University of Campania “Luigi Vanvitelli”, Naples, Italy; 2 Department of Psychiatry, Faculty of Medicine Ramathibodi Hospital, Mahidol University, Bangkok, Thailand; 3 Department of Psychiatry and Neuropsychology, School for Mental Health and Neuroscience, Maastricht University Medical Centre, Maastricht, The Netherlands; 4 Department of Psychiatry, UMC Utrecht Brain Centre, University Medical Centre Utrecht, Utrecht University, Utrecht, The Netherlands; 5 Department of Psychosis Studies, Institute of Psychiatry, Psychology & Neuroscience, King’s College London, London, UK; 6 Department of Psychiatry, Perelman School of Medicine, University of Pennsylvania, Philadelphia, PA USA; 7 Lifespan Brain Institute of the Children’s Hospital of Philadelphia and Penn Medicine, Philadelphia, PA USA; 8 Department of Child and Adolescent Psychiatry and Behavioral Science, Children’s Hospital of Philadelphia, Philadelphia, PA USA; 9 Department of Psychiatry, Yale University School of Medicine, New Haven, CT USA

**Keywords:** Psychosis, Psychosis

## Abstract

This study aimed to assess the independent and joint associations of genomic and exposomic liabilities for schizophrenia with distressing psychotic experiences (PEs) and their persistence in early adolescence. The Adolescent Brain and Cognitive Development Study data from children with European ancestry were used (*N* = 5122). The primary outcome was past-month distressing PEs at the 3-year follow-up. Secondary outcomes were distressing PEs at varying cutoffs of persistence. Multilevel logistic regression models were used to test the associations of binary modes (>75th percentile) of polygenic risk score for schizophrenia (PRS-SCZ_75_) and exposome score for schizophrenia (ES-SCZ_75_) on the outcomes. Relative excess risk due to interaction (RERI) calculation indicated additive interaction. When analyzed independently, PRS-SCZ_75_ was not significantly associated with past-month distressing PEs but with lifetime (OR 1.29 [95% CI 1.08, 1.53]) and repeating distressing PEs ≥2 waves (OR 1.34 [95% CI 1.08, 1.65]); whereas, ES-SCZ_75_ was consistently associated with all outcomes, with increasing strength of association as a function of PEs persistence (one wave: OR 2.77 [95% CI 2.31, 3.31]; two waves: OR 3.16 [95% CI 2.54, 3.93]; three waves: OR 3.93 [95% CI 2.86, 5.40]; four waves: OR 3.65 [95% CI 2.34, 5.70]). When considered jointly, ES-SCZ_75_ and PRS-SCZ_75_ did not additively interact to predict past-month distressing PEs but showed significant additive interactions for lifetime (RERI = 1.26 [95%CI 0.14, 2.38]) and repeating distressing PEs ≥2 waves (RERI = 1.79 [95%CI 0.35, 3.23]). Genomic and exposomic liabilities for schizophrenia were independently and jointly associated with distressing PEs and their persistence in early adolescence.

## Introduction

Psychotic experiences (PEs) are observed in around 10% of children and adolescents and increase the odds of having any mental and psychotic disorders by three- and four-fold, respectively^1,2^. Specifically, PEs causing distress (i.e., distressing PEs) are associated with developmental delay, neuroimaging findings such as reduced subcortical and cortical volumes, and suicidal behavior^3,4^. Despite the transient nature of most PEs, a recent meta-analysis showed that around one-third of people having PEs at baseline reported a second PE within a year^5^. The persistence of PEs, especially when distressing, is also linked with multiple mental disorders and greater impairments^4,5^. Given their clinical significance as a marker for later clinical syndromes, a better understanding of the etiology of distressing PEs as well as their persistence among youth is needed.

The shared heritability of PEs with psychotic disorders has long been discussed^6^. Genome-wide association studies (GWAS) have provided further evidence for shared genetic liability between PEs and schizophrenia^7,8^. In recent years, polygenic risk score (PRS), generated by summing the risk alleles weighted by their effect sizes derived from related GWAS, has been increasingly utilized to estimate an individual’s genetic predisposition to behavioral phenotypes and psychiatric disorders. Prior studies have shown that PRS for schizophrenia (PRS-SCZ) is associated with subclinical psychosis expression in children and adolescents^9–11^ as well as in adults^12^. However, since some researchers did not obtain similar results, probably due to differences in PRS-SCZ calculation and population samples, further investigations are required^13,14^.

Similar to genetic liability, an additive risk of environmental exposures has been shown to increase the odds of psychosis expression^15,16^. From the conceptualization of the “exposome”, a dense network of interdependent environmental exposures^17^, the exposome score for schizophrenia (ES-SCZ) has been developed using a predictive modeling approach^18^. ES-SCZ is calculated by summing the weighted risks of nine binary variables that represent well-established non-genetic risk factors for schizophrenia. Apart from its predictive ability for clinical psychosis^18^, ES-SCZ is also associated with schizotypal traits in siblings of patients with schizophrenia and healthy subjects, suggesting an interplay between genetic and environmental risks for schizophrenia across the psychosis continuum^12^.

To the best of our knowledge, no study has tested the influence of exposomic liability for schizophrenia on subclinical psychosis expression, both independently and jointly with genomic liability, using a prospective design. Moreover, most previous studies have rarely examined the persistence of distressing PEs despite their association with poor prognostic features^4,5^. Given these gaps, we aimed to assess the longitudinal associations of PRS-SCZ, ES-SCZ, and their interaction with distressing PEs along with their influences on symptom persistence, in a general population-based sample of young adolescents with European ancestry.

## Methods

### Participants

A sample of 11,876 participants was derived from the Adolescent Brain and Cognitive Development (ABCD) cohort, an ongoing multisite longitudinal study conducted in the United States to assess biological and behavioral development from early adolescence to young adulthood^19^. Physical health, neurocognition, mental health, substance use, environmental characteristics, and the usage of mobile technology are assessed annually through interviews with youth and their parents, while biospecimens are collected every year. Moreover, six-monthly assessments are performed by phone to receive updates in terms of mental health and substance use. The data from the baseline through the 3-year follow-up of the ABCD Data Release 5.1 were used (see Acknowledgements). Participants of European descent with good-quality genotyping data were included in the current analyses.

The ABCD Study was approved by the Centralized Institutional Review Board (IRB) of the University of California-San Diego and local research site IRBs^19^. Written informed consent and assent were provided by participating parents/caregivers and adolescents, respectively^19^.

### Variables

#### Psychotic experiences

PEs were assessed using the Prodromal Questionnaire-Brief Child Version (PQ-BC), a self-reported tool validated for the school-age population^20^. It comprises 21 items asking about PEs (e.g., unusual thought content, perceptual abnormality) in the past month. When adolescents reported the presence of any PEs, they were asked to rate the distress level related to the experience from 1 to 5. Consistent with previous research^11^, “distressing PEs” were regarded as present if at least one PE was rated 3 points or more; otherwise, absent. Past-month distressing PEs at the 2-year follow-up were used as the primary outcome. To explore the effects of risk exposure on the persistence of distressing PEs, four dichotomous variables were generated at varying degrees of persistence of distressing PEs from baseline to the 3-year follow-up as secondary outcomes: “lifetime distressing PEs” (i.e., presence of distressing PEs ≥ 1 wave), “repeating distressing PEs ≥ 2 waves” (i.e., presence of distressing PEs ≥ 2 waves), “repeating distressing PEs ≥ 3 waves” (i.e., presence of distressing PEs ≥ 3 waves), and “persisting distressing PEs” (i.e., distressing PEs in all 4 waves).

#### Polygenic risk score for schizophrenia

PRS-SCZ was constructed for each participant who passed the genetic and sample quality control. The pre-imputational quality control (Table S1), relatedness and ancestry distributions (Fig. S1 and Tables S2–S3), post-imputational data (Table S4) and principal components analysis for ancestry (Fig. S2–S5) are reported in detail in the online supplement. PRS-SCZ was built using data from the most recent schizophrenia GWASs (European subsample, i.e. 55.28% of the total ABCD sample) based on 53,386 cases and 77,258 controls^21^.

PRS_cs-auto_-SCZ^22^ was used to infer PRS generated using posterior SNP effect sizes, by placing a continuous shrinkage (cs) prior on SNP weights reported in the latest GWAS for schizophrenia^21^ and combined with an external linkage disequilibrium reference panel, such as the 1000 Genomes Project European Sample (https://github.com/getian107/PRScs). To compute posterior effect sizes, the default settings of PRS-cs-auto were used (See online supplement). After the calculation of posterior effect sizes, PRSs were calculated using ‘--score’ function and the SUM modifier in PLINK1.9. After the quality control, 742,011 variants were used for the PRS calculation. Besides PRS_cs-auto_-SCZ, we also calculated PRS_ice_-SCZ by the clumping and threshold methods using PRSice2^23^ for sensitivity analysis to verify the results.

#### Exposome score for Schizophrenia (ES-SCZ)

Following prior studies^12,18^, ES-SCZ was generated using nine environmental exposures: emotional neglect, physical neglect, emotional abuse, physical abuse, sexual abuse, cannabis use, winter birth, hearing impairment, and bullying. Variables in the ABCD dataset were carefully selected to generate each domain, aligning with the original ES-SCZ study^18^ and prior studies involving these risk factors in the ABCD dataset^24,25^. To ensure the antecedence of the exposure to the outcome measurement at the 3-year follow-up, ES-SCZ was calculated based on lifetime exposure to each environmental risk (0 = “absent”; 1 = “present”), using the information at baseline, 1-year follow-up, and 2-year follow-up. The variables used to construct each exposome factor are provided in Table S7, and the detailed construction of ES-SCZ is described in the online supplement. Briefly, emotional neglect was derived from five items of the Child Report of Parent Behaviors Inventory^26^, asking children to rate their perceived levels of parents’ warmth and closeness. Physical neglect was regarded as present if children had experienced any defined levels of food shortage, history of maternal or paternal misuse of alcohol, and poor parental supervision, as reported in three ABCD surveys assessing parental demographics, parental monitoring, and family history. Emotional abuse, physical abuse, and sexual abuse were derived from the PTSD module of parent-reported Kiddie Schedule for Affective Disorders and Schizophrenia for DSM-5^27^. Cannabis use was derived from multiple assessments of frequency of use, craving levels, and withdrawal symptoms, based on children’s and parents’ reports from baseline to 2-year follow-up, including mid-year telephone interviews. Winter birth was determined based on the precise date of the interview at baseline and the child’s age in months at the time of the interview. Hearing impairment was derived from the Parent Medical History Questionnaire, asking if the child had ever been to a doctor for hearing problems. Bullying was derived from the Parent Diagnostic Interview for DSM-5 Background Items, asking if the children had any problems with bullying at school or in the neighborhood. Finally, the weighted risks (i.e., each exposure multiplied by its log odds for schizophrenia) of the nine exposures were summed up and added by 2 for ease of interpretation (see the online supplement)^18^.

#### Covariates

Age in years at the time of PE assessment, sex, baseline family income, and highest parental education were included as covariates. Model 1 analysis comprised only age and sex, while all covariates were included in Model 2. The detailed descriptions of the covariates are presented in Table S7 in the online supplement.

### Statistical analysis

Stata, version 16.1 was used for statistical analyses^28^. Samples with missing primary outcome data or exposome variables were excluded from the study, and those with missing covariates were excluded from related analyses. The number of missing observations is reported in Table S8 in the online supplement. Similar to prior studies^12,29^, PRS-SCZ and ES-SCZ were dichotomized at the 75th percentile (hereafter PRS-SCZ_75_ and ES-SCZ_75_, respectively).

#### Main analyses

The main effects of PRS_cs-auto_-SCZ_75_ and ES-SCZ_75_ at the 2-year follow-up on distressing PE at the 3-year follow-up were tested in separate models using multilevel logistic regression analysis with random intercepts for collection sites and family. Past-month distressing PEs at the 3-year follow-up were used as the primary outcome to test the prospective association of the ES-SCZ, adjusted for distressing PEs from prior waves (i.e., baseline to 2-year follow-up). Lifetime distressing PEs, repeating distressing PEs ≥ 2 waves, repeating distressing PEs ≥ 3 waves, and persisting distressing PEs were used as secondary outcomes to explore the influences of PRS-SCZ and ES-SCZ on varying degrees of persistence of distressing PEs. Consistent with previous work^12,29^, additive models were used to test the interaction effects between PRS_cs-auto_-SCZ_75_ and ES-SCZ_75_. As an indicator of the departure from additivity, relative excess risk due to interaction (RERI)^30^ was calculated through the delta method^31^ using the Stata “nlcom” postestimation command. A RERI greater than zero reflects additional risk beyond the sum of the independent risks of genetic and environmental factors. Similar to prior studies^11,32^, the models were adjusted for two sets of covariates: Model 1) age and sex; Model 2) age, sex, family income, and parental education. Analyses including PRS_cs-auto_-SCZ_75_ were additionally adjusted for the first ten ancestrally informative genetic principal components. The nominal significance threshold was set to *p* < 0.05 for the primary outcome. As the secondary outcomes were of explorative nature, we did not apply a correction for multiple testing^33^.

#### Sensitivity analyses

As PRS-cs-auto is a relatively new method, PRS_ice_-SCZ75, generated by the clumping and thresholding method, was used to test the main genetic effect and its interaction with the environment on distressing PEs to ensure comparability with existing studies.

## Results

### Sample characteristics

Table 1 shows the baseline sociodemographic characteristics of the sample included in the main analyses (*N* = 5122) and the exposure frequencies at the 2-year follow-up. At the 3-year follow-up, participants were 12.9 years (SD = 0.65, range 11.5–14.6 years), with 11.0% (*N* = 561) having distressing PEs in the past month. Lifetime (≥1 wave), repeating ≥ 2 waves, repeating ≥ 3 waves, and persisting (all 4 waves) distressing PEs were endorsed by 1751 (34.2%), 741 (14.5%), 289 (5.6%), and 98 (1.9%) participants, respectively.

**Table 1:** Sample characteristics at baseline and lifetime exposure through the 2-year follow-up.

Characteristics at baseline (*N* = 5122)	Mean	SD
Age (years)	9.93	0.63
Parental educational attainment (years)	18.2	1.7
	***N***	**%**
Male	2721	53.1
Family income		
<$5000	28	0.6
$5000-$11,999	34	0.7
$12,000-$15,999	30	0.6
$16,000-$24,999	78	1.6
$25,000-$34,999	127	2.6
$35,000-$49,999	268	5.5
$50,000-$74,999	668	13.6
$75,000-$99,999	841	17.1
$100,000-$199,999	2058	41.9
≥ $200,000	780	15.9
**Lifetime exposure through the 2-year follow-up**	***n***	**%**
Physical abuse	38	0.7
Emotional abuse	52	1.0
Sexual abuse	126	2.5
Physical neglect	962	18.8
Emotional neglect	118	2.3
Bullying	1486	29.0
Winter birth	1571	30.7
Hearing impairment	365	7.1
Cannabis use	8	0.2

### Main effects of PRS-SCZ

**Fig. 1:**
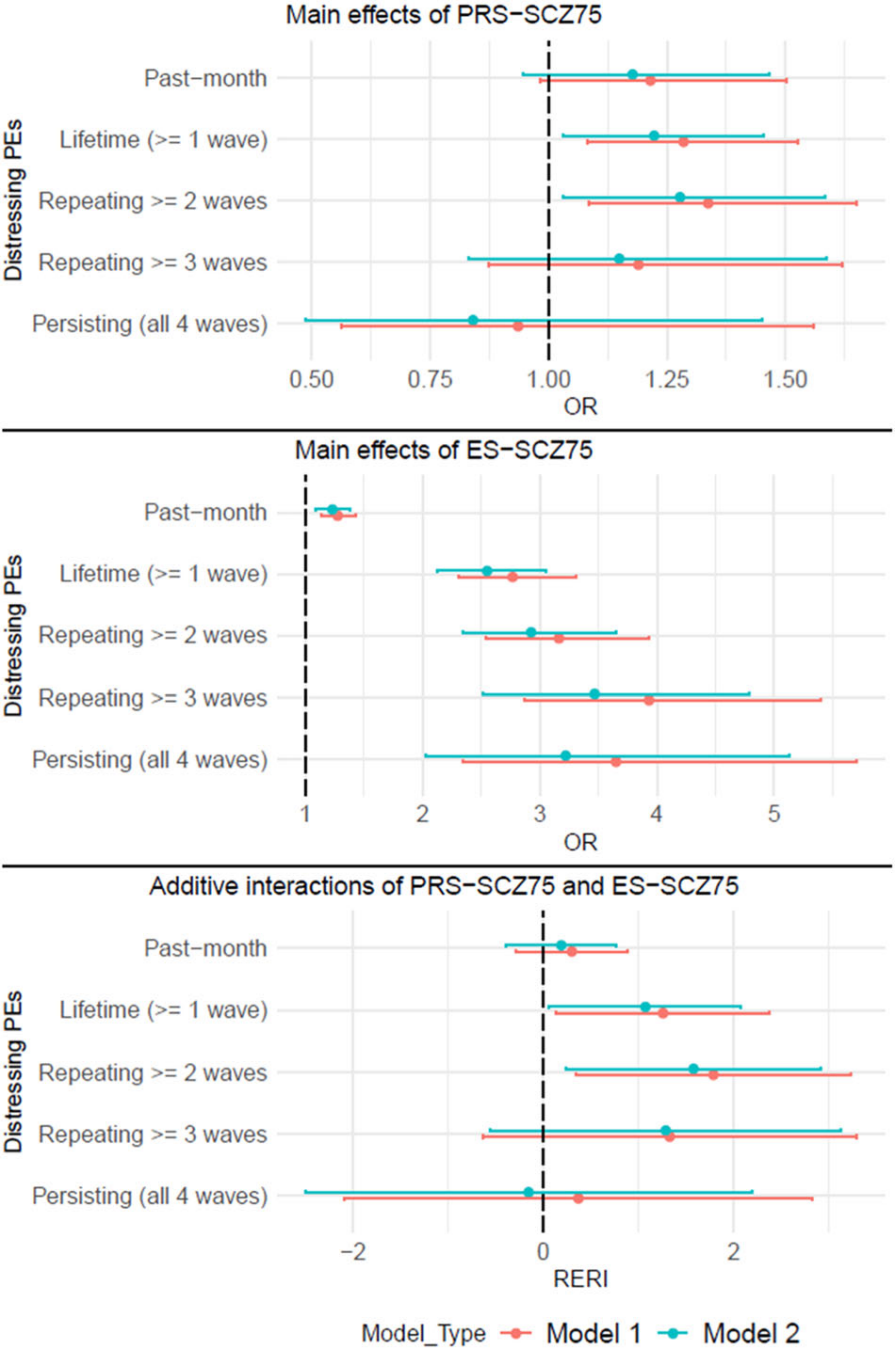
Visualization of main and interacting associations of PRS_cs-auto_-SCZ_75_ and ES-SCZ_75_ at the 2-year follow-up on distressing PEs with varying degrees of symptom persistence at the 3-year follow-up. Model 1: adjusted for age and sex; Model 2: adjusted for age, sex, family income, and parental education; Upper panel: Main effects of PRS_cs-auto_-SCZ_75_ (additionally adjusted for 10 PCs); Middle panel: Main effects of ES-SCZ_75_ (additionally adjusted for distressing PEs up to the 2-year follow-up for past-month distressing PEs); Lower panel: Additive interaction between PRS_cs-auto_-SCZ_75_ and ES-SCZ_75_ (additionally adjusted for 10 PCs for all outcomes and distressing PEs up to the 2-year follow-up for past-month distressing PEs). OR, odds ratio; RERI, relative excess risk due to interaction.

Primary analysis revealed that PRS_cs-auto_-SCZ_75_ was not significantly associated with past-month distressing PEs at the 3-year follow-up (Table 2). However, secondary analyses showed that PRS_cs-auto_-SCZ_75_ was significantly associated with lifetime distressing PEs (Model 1: OR 1.29 [95% CI 1.08, 1.53], *p* = 0.004; Model 2: OR 1.22 [95% CI 1.03, 1.45], *p* = 0.022) and repeating distressing PEs ≥ 2 waves (Model 1: OR 1.34 [95% CI 1.08, 1.65], *p* = 0.007; Model 2: OR 1.28 [95% CI 1.03, 1.58], *p* = 0.025) but not with repeating distressing PEs ≥ 3 waves and persisting distressing PEs at the 3-year follow-up (Table 2 & Fig. 1A). Sensitivity analysis confirmed significant associations of PRS_ice_-SCZ_75_ with lifetime distressing PEs only when adjusted for age and sex (OR 1.22 [95% CI 1.02, 1.45], *p* = 0.025), but not when additionally adjusted for family income and parental education (OR 1.18 [95% CI 0.99, 1.40], *p* = 0.066). No other significant associations in the main analyses were confirmed (Table S9 in the online supplement).

**Table 2:** Main associations of PRS_cs-auto_-SCZ_75_ with distressing PEs at the 3-year follow-up.

Distressing PEs	Model 1 (*N* = 5,122)			Model 2 (*N* = 4,912)		
	OR	95% CI	*p*-value	OR	95% CI	*p*-value
Past-month	1.22	0.98 to 1.50	0.072	1.18	0.95 to 1.47	0.143
Lifetime (≥ 1 wave)	1.29	1.08 to 1.53	**0.004**	1.22	1.03 to 1.45	**0.022**
Repeating (≥ 2 waves)	1.34	1.08 to 1.65	**0.007**	1.28	1.03 to 1.58	**0.025**
Repeating (≥ 3 waves)	1.19	0.87 to 1.62	0.269	1.15	0.83 to 1.59	0.397
Persisting (all 4 waves)	0.94	0.56 to 1.56	0.799	0.84	0.49 to 1.45	0.534

Model 1: Adjusted for age and sex; Model 2: Adjusted for age, sex, family income, and parental education. *p*-values < 0.05 were bolded. *PEs* psychotic experiences, *OR* odds ratio, *CI* confidence interval.

### Main effects of ES-SCZ

ES-SCZ_75_ at the 2-year follow-up was significantly associated with past-month distressing PEs (Model 1: OR 1.45 [95% CI 1.19, 1.75], *p* < 0.001; Model 2: OR 1.37 [95% CI 1.12, 1.68], *p* = 0.002), as well as with all secondary outcomes at the 3-year follow-up: lifetime distressing PEs (Model 1: OR 2.77 [95% CI 2.31, 3.31], *p* < 0.001; Model 2: OR 2.55 [95% CI 2.13, 3.05], *p* < 0.001), repeating distressing PEs ≥ 2 waves (Model 1: OR 3.16 [95% CI 2.54, 3.93], *p* < 0.001; Model 2: OR 2.93 [95% CI 2.35, 3.65], *p* < 0.001), repeating distressing PEs ≥ 3 waves (Model 1: OR 3.93 [95% CI 2.86, 5.40], *p* < 0.001; Model 2: OR 3.47 [95% CI 2.51, 4.78], *p* < 0.001), and persisting distressing PEs (Model 1: OR 3.65 [95% CI 2.34, 5.70], *p* < 0.001; Model 2: OR 3.22 [95% CI 2.02, 5.13], *p* < 0.001) (Table 3). As shown in Fig. 1B, the effects of ES-SCZ_75_ on distressing PEs tended to be stronger when the experiences become more persistent.

**Table 3:** Main associations of ES-SCZ_75_ with distressing PEs at the 3-year follow-up.

Distressing PEs	Model 1 (*N* = 5,122)			Model 2 (*N* = 4,912)		
	OR	95% CI	*p*-value	OR	95% CI	*p*-value
^a^Past-month	1.45	1.19 to 1.75	**<0.001**	1.37	1.12 to 1.68	**0.002**
Lifetime (≥1 wave)	2.77	2.31 to 3.31	**<0.001**	2.55	2.13 to 3.05	**<0.001**
Repeating (≥2 waves)	3.16	2.54 to 3.93	**<0.001**	2.93	2.35 to 3.65	**<0.001**
Repeating (≥3 waves)	3.93	2.86 to 5.40	**<0.001**	3.47	2.51 to 4.78	**<0.001**
Persisting (all 4 waves)	3.65	2.34 to 5.70	**<0.001**	3.22	2.02 to 5.13	**<0.001**

Model 1: Adjusted for age and sex; Model 2: Adjusted for age, sex, family income, and parental education. *p*-values < 0.05 were bolded. *PEs* psychotic experiences, *OR* odds ratio, *CI* confidence interval.

^a^Additionally adjusted for distressing PEs up to the 2-year-follow-up

### Joint effects of PRS-SCZ and ES-SCZ

When considered jointly, the isolated genetic risk state (i.e., PRS_cs-auto_-SCZ_75_ = 1 & ES-SCZ_75_ = 0) was not associated with past-month distressing PEs at the 3-year follow-up (Model 1: OR 1.02 [95% CI 0.78, 1.35], *p* = 0.868; Model 2: OR 1.04 [95% CI 0.79, 1.38], *p* = 0.759), whereas the isolated exposomic risk state (i.e., PRS_cs-auto_-SCZ_75_ = 0 & ES-SCZ_75_ = 1) significantly increased the odds of having past-month distressing PEs by 33–38% (Model 1: OR 1.38 [95% CI 1.10, 1.73], *p* = 0.005; Model 2: OR 1.33 [95% CI 1.06, 1.69], *p* = 0.016) (Table 4). Even though having both genetic and exposomic risk states (i.e., PRS_cs-auto_-SCZ_75_ = 1 & ES-SCZ_75_ = 1) was also associated with past-month distressing PEs (Model 1: OR 1.71 [95% CI 1.25, 2.34], *p* = 0.001; Model 2: OR 1.57 [95% CI 1.13, 2.17], *p* = 0.007), the OR did not significantly increase beyond the sum of the ORs for either genetic or exposomic risk state alone, indicating a null additive interaction between PRS_cs-auto_-SCZ_75_ and ES-SCZ_75_ on past-month distressing PEs (Model 1: RERI 0.30 [95% CI −0.29, 0.89], *p* = 0.312; Model 2: RERI 0.19 [95% CI −0.39, 0.77], *p* = 0.522) (Table 4 & Fig. 1C).

**Table 4:** Additive interaction between PRS_cs-auto_-SCZ_75_ and ES-SCZ_75_ at the 2-year follow-up on distressing PEs at the 3-year follow-up.

Distressing PEs		Model 1 (*N* = 5,122)			Model 2 (*N* = 4,912)		
		PRS_cs-auto_-SCZ_75_ = 0 OR (95% CI)	PRS_cs-auto_-SCZ_75_ = 1 OR (95% CI)	RERI (95% CI)	PRS_cs-auto_-SCZ_75_ = 0 OR (95% CI)	PRS_cs-auto_-SCZ_75_ = 1 OR (95% CI)	RERI (95% CI)
^a^Past-month (3-year follow-up)	ES-SCZ_75_ = 0	1.0	1.02 (0.78 to 1.35) *p* = 0.868	0.30 (−0.29 to 0.89) *p* = 0.312	1.0	1.04 (0.79 to 1.38) *p* = 0.759	0.19 (−0.39 to 0.77) *p* = 0.522
	ES-SCZ_75_ = 1	1.38 (1.10 to 1.73) ***p*** = **0.005**	1.71 (1.25 to 2.34) ***p*** = **0.001**		1.33 (1.06 to 1.69) ***p*** = **0.016**	1.57 (1.13 to 2.17) ***p*** = **0.007**	
Lifetime (≥ 1 wave)	ES-SCZ_75_ = 0	1.0	1.17 (0.95 to 1.44) *p* = 0.142	1.26 (0.14 to 2.38) ***p*** = **0.027**	1.0	1.11 (0.90 to 1.37) *p* = 0.322	1.07 (0.06 to 2.08) ***p*** = **0.037**
	ES-SCZ_75_ = 1	2.51 (2.06 to 3.05) ***p*** < **0.001**	3.93 (2.92 to 5.29) ***p*** < **0.001**		2.31 (1.90 to 2.82) ***p*** < **0.001**	3.50 (2.59 to 4.72) ***p*** < **0.001**	
Repeating (≥ 2 waves)	ES-SCZ_75_ = 0	1.0	1.13 (0.86 to 1.49) *p* = 0.391	1.79 (0.35 to 3.23) ***p*** = **0.015**	1.0	1.08 (0.81 to 1.43) *p* = 0.589	1.58 (0.24 to 2.92) ***p*** = **0.021**
	ES-SCZ_75_ = 1	2.77 (2.18 to 3.52) ***p*** < **0.001**	4.69 (3.35 to 6.57) ***p*** < **0.001**		2.58 (2.02 to 3.29) ***p*** < **0.001**	4.24 (3.01 to 5.97) ***p*** < **0.001**	
Repeating (≥ 3 waves)	ES-SCZ_75_ = 0	1.0	1.01 (0.65 to 1.57) *p* = 0.955	1.33 (−0.64 to 3.29) *p* = 0.185	1.0	0.95 (0.60 to 1.51) *p* = 0.843	1.29 (−0.56 to 3.13) *p* = 0.171
	ES-SCZ_75_ = 1	3.42 (2.44 to 4.80) ***p*** < **0.001**	4.76 (3.00 to 7.57) ***p*** < **0.001**		3.03 (2.14 to 4.30) ***p*** < **0.001**	4.28 (2.65 to 6.90) ***p*** < **0.001**	
Persisting (all 4 waves)	ES-SCZ_75_ = 0	1.0	0.82 (0.38 to 1.77) *p* = 0.619	0.37 (−2.09 to 2.83) *p* = 0.770	1.0	0.83 (0.38 to 1.82) *p* = 0.635	−0.15 (−2.50 to 2.19) *p* = 0.898
	ES-SCZ_75_ = 1	3.48 (2.07 to 5.83) ***p*** < **0.001**	3.67 (1.82 to 7.40) ***p*** < **0.001**		3.31 (1.90 to 5.75) ***p*** < **0.001**	2.98 (1.37 to 6.45) ***p*** = 0**.006**	

Model 1: Adjusted for age and sex; Model 2: Adjusted for age, sex, family income, and parental education. *P*-values < 0.05 were bolded.

^a^Additionally adjusted for distressing PEs up to 2-year-follow-up.

**Fig. 2:**
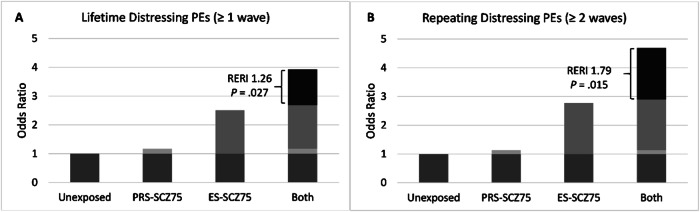
Additive interaction effects. Additive interaction between PRS_cs-auto_-SCZ_75_ and ES-SCZ_75_ on lifetime distressing PEs (**A**) and repeating distressing PEs ≥ 2 waves (**B**), adjusted for age, sex, 10 PCs (Model 1). OR, odds ratio; RERI, relative excess risk due to interaction.

Similarly, the isolated genetic risk state (i.e., PRS_cs-auto_-SCZ_75_ = 1 & ES-SCZ_75_ = 0) was not associated with any secondary outcomes, while the isolated exposomic risk state (i.e., PRS_cs-auto_-SCZ_75_ = 0 & ES-SCZ_75_ = 1) significantly increased the odds of having distressing PEs, with rising ORs for more recurrent distressing PEs across a lifetime (Table 4). Having both genetic and exposomic risk states (i.e., PRS_cs-auto_-SCZ_75_ = 1 & ES-SCZ_75_ = 1) significantly increased the odds of lifetime distressing PEs ≥ 1 and repeating distressing PEs ≥ 2 waves beyond the sum of the ORs of having either risk state alone by 107–126% (Model 1: RERI 1.26 [95% CI 0.14, 2.38], *p* = 0.027; Model 2: RERI 1.07 [95% CI 0.06, 2.08], *p* = 0.037) (Fig. 2A) and 158–179% (Model 1: RERI 1.79 [95% CI 0.35, 3.23], *p* = 0.015; Model 2: RERI 1.58 [95% CI 0.24, 2.92], *p* = 0.021) (Fig. 2B), respectively. However, no significant additive interactions between PRS_cs-auto_-SCZ_75_ and ES-SCZ_75_ were detected for repeating distressing PEs ≥ 3 waves and persisting distressing PEs at the 3-year follow-up (Table 4). Sensitivity analyses using PRS_ice_-SCZ_75_ did not confirm the significant additive interactions (Table S10 in the online supplement).

## Discussion

To our knowledge, this is the first longitudinal investigation of the independent and joint associations of genomic and exposomic liabilities for schizophrenia with distressing PEs and their persistence in early adolescence. Our findings show that PRS-SCZ was associated with distressing PEs and additively interacted with ES-SCZ in predicting lifetime distressing PEs reported in at least one or two of four annual assessments. We also found a significant association between ES-SCZ in the previous year and distressing PEs reported in the following year, suggesting a prospective association of ES-SCZ and distressing PEs. Our secondary analyses revealed that the strength of the associations of ES-SCZ with distressing PEs increased as a function of the persistence of the experiences.

A significant association between PRS-SCZ and distressing PEs confirms the notion that PEs share genetic liability with schizophrenia across the psychosis continuum^6^. This aligns with prior studies on PRS-SCZ and subclinical psychosis expression in late adolescence^9,10^ and adulthood^12^ and is consistent with a cross-sectional analysis of the ABCD dataset at baseline^11^. However, we did not find an association between PRS-SCZ and distressing PEs that repeated for three or more waves. One possible explanation is that the lower number of participants with more persistent distressing PEs might not have had enough statistical power to detect a small effect size of the genetic influence on low-prevalance outcomes. Similarly, previous studies reporting a non-significant association between PRS-SCZ and PEs also appeared to suffer from issues that might reduce statistical power: relatively small sample sizes (<2500), less powerful discovery GWAS, and ethnically mixed populations^7,14^. Alternatively, the non-significant main effect of PRS-SCZ despite the significant main effect of ES-SCZ on repeating distressing PEs ≥ 3 waves might reflect a higher contribution of environmental risk compared to genetic risk factors for more persisting distressing PEs. Considering that the genetic overlap between SCZ and PEs was greater in adults compared to adolescents^34^, and the prevalence of repeating distressing PEs would be increasing over time, a replication study when the ABCD cohort reaches early adulthood might clarify this and provide a deeper insight into the age-dependent relationship between PRS-SCZ and PEs persistence.

Compared to PRS-SCZ, ES-SCZ appeared to be more strongly associated with distressing PEs across all outcomes and models. This finding conforms with previous research showing that environmental risk factors for schizophrenia explain more of the variance in subthreshold positive symptoms than PRS-SCZ^35^. Furthermore, we demonstrated that the magnitude of the association of ES-SCZ with distressing PEs increased as a function of symptom persistence. This is in line with the prior analysis of two large prospective general population cohorts, showing that the persistence rate of PEs progressively increases with greater environmental risk^36^. As the persistence of distressing PEs is considered a predictor of not only future psychotic disorders but also other psychopathology^2,5^, our findings suggest that modifying environmental risk could be a promising strategy to prevent psychopathology among youth.

When considering the effects of PRS-SCZ and ES-SCZ jointly, we found that the isolated exposomic risk state was associated with distressing PEs of all definitions, with increasing ORs as a function of PEs persistence, similar to the findings from the independent ES-SCZ analysis. On the other hand, no significant associations were found between the isolated genetic risk state and distressing PEs of any definition, despite the significant associations of PRS-SCZ with lifetime distressing PEs (≥ 1 wave) and repeating distressing PEs ≥ 2 waves in the independent PRS-SCZ analysis. These findings indicate that PRS-SCZ has a weaker effect on distressing PEs, which loses its significance when considered jointly with ES-SCZ. Interestingly, having both genetic and exposomic risk states significantly increased the odds of lifetime distressing PEs (≥1 wave) and repeating distressing PEs ≥ 2 waves beyond the sum of the ORs for having either risk state alone, suggesting positive additive interactions between PRS-SCZ and ES-SCZ for these outcomes. However, the sensitivity analysis using the PRS-ice did not confirm the additive interactions with ES-PRS, probably due to a lower performance of PRS-ice compared to PRS-cs-auto^22^. Consistent with our main analyses, a previous study reported a significant additive interaction between PRS-SCZ and ES-SCZ in predicting schizotypal traits among healthy adults and unaffected siblings of patients with schizophrenia^12^. Similarly, evidence from two large adolescent cohorts revealed that the heritability of PEs decreases as the environmental risk load increases^37^, supporting a stronger influence of the environment over genetic factors and their interplay in subclinical psychosis. Conversely, an analysis of a Brazilian children cohort revealed neither main nor interacting effects of polygenic and poly-environmental risk scores for schizophrenia on PEs, which might be partly explained by the population admixture of the Brazilian sample^14^. Although more studies using larger datasets are required to rule out the possibility of the low statistical power in genetic risk analysis, our findings collectively support the synergism between genetic and exposomic risks for schizophrenia in determining subthreshold psychosis expression.

Prior research from the ABCD study has shown that children with distressing PEs who presented symptoms at least twice across three waves exhibited the greatest impairment compared to those with transient or non-distressing PEs^4^. Together with this evidence, our findings corroborate the psychosis proneness-persistence-impairment model^38^. Guided by meta-analytical evidence, the model suggests that, despite the transient nature of most PEs, these PEs could abnormally persist and lead to subsequent impairment if the person is additionally exposed to environmental exposures, which also interact with the individual genetic background^38^. Indeed, empirical evidence shows that the persistent trajectory of PEs is associated with secondary distress and strongly predicts a transition from subclinical to clinical psychosis over time^39^. Considering that the prevalence of PEs and their persistence peak in adolescence^5^, screening for psychosis proneness with subsequent early intervention aimed at reducing environmental risk exposure could be an effective strategy to prevent the persistence of distressing PEs with subsequent impairment and eventual development of clinical psychosis.

Our research should be considered in light of some limitations. First, PEs were assessed using a self-reported questionnaire that could be subject to recall bias^5^. However, the validity of a self-screening questionnaire for PEs has been tested against clinical interviews in adolescents, demonstrating acceptable sensitivity and specificity^40^. More specific to the ABCD study, Karcher et al.^41^ showed that the association between parent-rated (through Child Behavior Checklist – psychosis) and child-rated (using the PQ-BC) PEs was weak, with the latter being more associated with known psychosis risk factors than the former, supporting the validity of the child’s report. Nonetheless, the one-month timeframe inquired by PQ-BC might limit the detection of PEs that fall outside the assessment window, potentially resulting in an underestimation of the association.

Second, since the recurrence and distressing quality of PEs have been shown to be linked to subsequent clinical outcomes^4,5^, the current analysis mainly focused on these two characteristics and did not examine other aspects of PEs. Therefore, future research might explore other severity indicators of PEs, such as the total number of the experiences and the degree of associated distress, to determine whether these characteristics also show a significant association and a dose-response relationship with the genomic and exposomic risk loads for schizophrenia, similar to what we found for the persistence of distressing PEs.

Third, we restricted our analyses to European subpopulations to ensure the optimal performance of PRS-SCZ derived from the most recent GWAS of the same ancestries^21,42^. This approach might limit the generalizability of our findings to non-European ancestries and decrease the statistical power of analyses for less prevalent outcomes or small effect sizes. Indeed, we failed to detect the main effects of PRS-SCZ and its interaction with ES-SCZ for more persistent distressing PEs, even though the larger main effects of ES-SCZ on distressing PEs could be confirmed at all degrees of persistence across adjusted models and sensitivity analyses. Therefore, a replication analysis using a large non-European cohort is required to improve the generalizability of the findings and ensure adequate power to detect potentially smaller influences of genetic factors and their interaction with environmental exposure in predicting the persistence of distressing PEs.

Fourth, it should be noted that the temporal precedence of the exposomic risk could be ascertained only for past-month distressing PEs and persisting distressing PEs (all 4 waves); therefore, the temporal relationship required to infer the causality of ES-SCZ for the other secondary outcomes cannot be assumed. Moreover, preceding distressing PEs might subsequently increase the propensity for exposure to certain environmental risks, such as cannabis use^43^, resulting in overestimated ORs of ES-SCZ for distressing PEs across lifetime. However, among the exposures, cannabis use was the least prevalent (0.2%) in this young adolescent sample, whereas being bullied was the most prevalent (29%) and also contributed the highest risk load to ES-SCZ. As prior evidence has shown that PEs do not predict later victimization by bullying in early adolescence^44^, such an overestimation of the effects of ES-SCZ on PEs persistence is likely to be of marginal concern. Nevertheless, considering that ES-SCZ is mainly constructed from environmental risks that would become accumulated over time, such as childhood adverse experiences and cannabis use, it is likely that the predictive power of ES-SCZ for psychopathology will increase in the follow-up cohort in late adolescence or early adulthood.

Lastly, although the predictive capacity of ES-SCZ has been well-tested in both clinical and non-clinical populations^12,18^, increasing the availability of comprehensive data resources linked to electronic health records and geolocated data in the future could allow for improving ES-SCZ with additional exposures. Moreover, integrating multiple sources of information to more accurately determine the environmental risk would complement our findings, which primarily relied on parent and child reports.

In conclusion, our findings highlight the shared genomic and exposomic etiology between subthreshold PEs and clinical psychosis expression, as well as their interactions underlying the persistence of distressing PEs. Although our findings require further replication, public health policy reforms aimed at minimizing environmental risk among young adolescents, particularly those with increased genetic liability for schizophrenia, could be a promising strategy to improve the population's mental well-being by preventing the progression of subclinical psychosis to clinically significant psychopathology.

## Supplementary information

SUPPLEMENTARY MATERIAL

## Data Availability

Data used in the preparation of this article were obtained from publicly available data from the Adolescent Brain Cognitive Development Study (https://abcdstudy.org), held in the National Institute of Mental Health Data Archive.
